# Single-staged Bilateral Revision Knee Prosthesis Results

**DOI:** 10.7759/cureus.4699

**Published:** 2019-05-21

**Authors:** Turan Cihan Dülgeroğlu, Suleyman Kozlu, Nihat Demirhan Demirkıran

**Affiliations:** 1 Orthopaedics and Traumatology, Kütahya Medical Sciences University Hospital, Kütahya, TUR; 2 Orthopaedics, Kütahya Health Sciences University School of Medicine, Kütahya, TUR

**Keywords:** bilateral revision, revision knee atrhroplasty, single staged, loosening

## Abstract

Total knee arthroplasty (TKA) can become impaired in the functionality of the bone-prosthetic unit for various reasons, thereby leading to prosthetic loosening. For patients with bilateral aseptic loosening, revision knee arthroplasty surgery is usually performed in different sessions.

Today, with developing anesthetic techniques, the patients' intraoperative and postoperative additional medical treatment needs are reduced; there is a reduction in complication rates too. Complications such as laryngospasm, bronchospasm, malignant hyperthermia, respiratory depression, postoperative delirium, or cognitive dysfunction can be seen. In the postoperative period, patient satisfaction, with adequate pain control, makes the rehabilitation of the knee is easier and shortens the duration of hospital stay.

In addition, the risks of complications such as deep venous thrombosis, pulmonary embolism, pneumonia, and urinary retention are decreased with early rehabilitation, preventing the development of arthrofibrosis. Maximum recovery in the early postoperative period may be possible with the early recovery of movement.

Between the years 2017 and 2018, patients admitted to our hospital for bilateral TKA application due to bilateral aseptic loosening and the early results of the application of bilateral revision TKA in one session with four selected patients are compared according to the requirements for blood transfusion and overall costs. Patient selection was shared with the anesthesiologist and the decision to continue bilaterally was made in the intraoperative assessment. In patients who did not develop any pathologies in the initial operation, the second operation was performed, where the risks of the second operation were not taken into account.

As a result, we conclude that bilateral revision TKA application on correct patient selection is a surgical procedure that can be performed safely by an experienced team.

## Introduction

Osteoarthritis (OA) is the most common cause of functional limitations and loss of movement in the elderly patient group [1-2]. The total knee arthroplasty (TKA) procedure is a satisfying and frequently preferred treatment option for knee osteoarthritis [3]. Bilateral knee OA has been reported in 19.7% to as high as 49.5% of patients in various studies [4-5]. Patients with both knees affected by the disease may also benefit from bilateral TKA surgeries. However, as the number of TKAs rises, the need for revision surgeries also increases concurrently. The leading cause of revision is aseptic component loosening [6].

A bilateral aseptic loosening of TKA may be treated in either each knee by two separate surgeries or simultaneously in a single procedure. Both treatment options have their own advantages. Although there have been several publications comparing two-staged and simultaneous bilateral primary TKA applications, only a few studies have evaluated the outcomes of simultaneous bilateral revision TKA surgeries [7-8].

Here, we present four patients who underwent a single-staged bilateral revision TKA for aseptic loosening. We report the perioperative blood transfusion rates, cost analysis results, and functional outcomes at the one-year follow-up.

## Case presentation

All surgeries were performed under pneumatic tourniquets. An anterior longitudinal skin incision and a medial parapatellar arthrotomy were performed. Antibiotic prophylaxis was initiated by intravenous 1 g cefazolin and 500 mg levofloxacin 30 minutes prior to the operation and continued at a dose of 1 g three times a day for three days. All patients underwent anesthesia with spinal plus epidural catheterization, except for one patient who underwent general anesthesia due to the difficulty of the application (previous lumbar region surgery).

After removing the old prosthesis, the patients underwent antibiotic cementing and revision arthroplasty with the Vanguard Revision Knee System (Zimmer Biomet, Inc, IN, US). At the end of the first knee, the second knee was started after the patient's vital signs and oxygen saturation were observed and evaluated by the anesthesiologist. In the meantime, the second knee was not started until the first knee was fully closed. The tourniquet was opened after the skin was closed and a compressive bandage was applied. All of the phases were repeated for the other knee while the shirts and gloves of the entire surgical team were replaced in the transition phase. A hemovac drain was routinely used and was taken out in all patients 24 hours after surgery.

**Figure 1:**
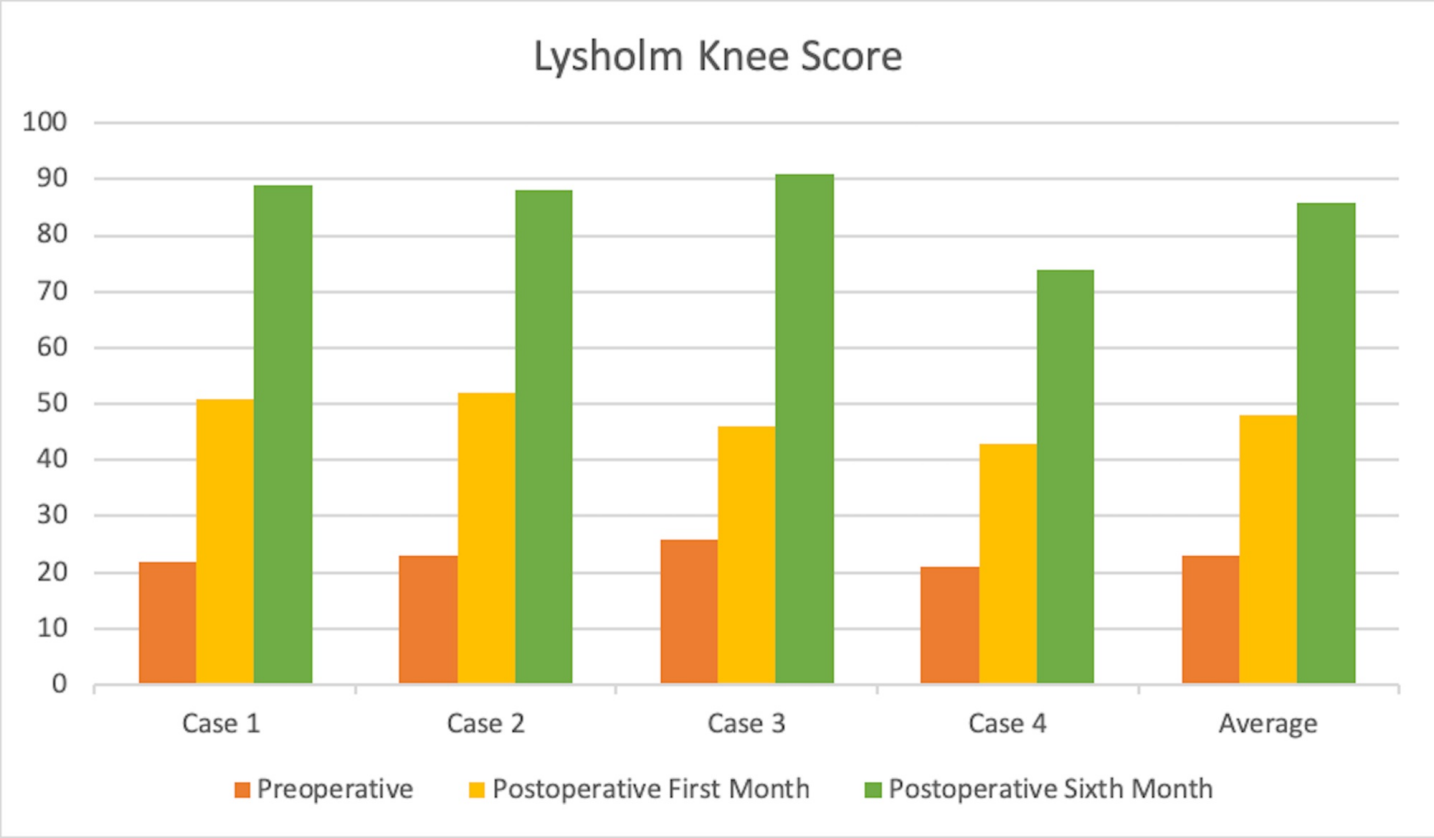
Lysholm Knee Scores of Our Cases

All patients were able to flex their knees to 110 degrees within a month, which was done in increments. The Lysholm knee scores of all four patients improved postoperatively at the first and sixth-month follow-ups (Figure 1).

In the early postoperative six months of our patients, there were no signs of infection and it was observed that the healing of the wound areas was complete. The difference in the preoperative and postoperative hemoglobin levels, the need for blood transfusion, and the hospital costs (excluding the price of prosthetic) was recorded and is shown in Table 1.

**Table 1:** Hospital Data of Cases

PATIENT	HOSPITAL TIME (day)	INTRODUCTION - EXCLUSION HEMOGLOBIN DIFFERENCES (g/dl)	BLOOD TRANSFUSION NEEDS (unıt)	HOSPITAL COST ( EXCEPT FOR PROSTHESIS ) (Turkish lira)
1	6	2.7	2	2890
2	10	3.0	3	4430
3	7	2.6	1	2930
4	9	3.2	2	3450
AVERAGE	8	2.8	2	3425

Case 1

**Figure 2:**
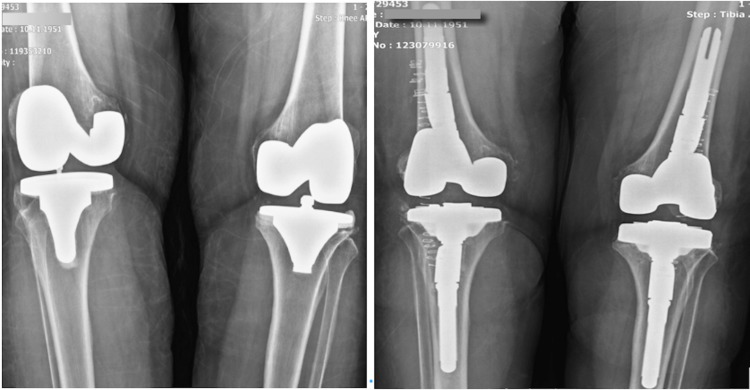
Case 1 Preoperative and Postoperative Knee X-Ray

In our first case, a 67-year-old female patient presented with aseptic loosening findings on both knees. The patient was operated on the right knee five years ago and on the left knee four years ago. The operation lasted about 160 minutes. One unit of erythrocyte suspension (ES) replacement was given in response to bleeding of the patient, who did not develop any complications during the operation. Patient-controlled analgesia (PCA) application was performed via an epidural catheter for postoperative pain. An intra-articular transamine application was performed. In the first 24 hours, the patient needed one unit of ES replacement (Figure 2).

Case 2

**Figure 3:**
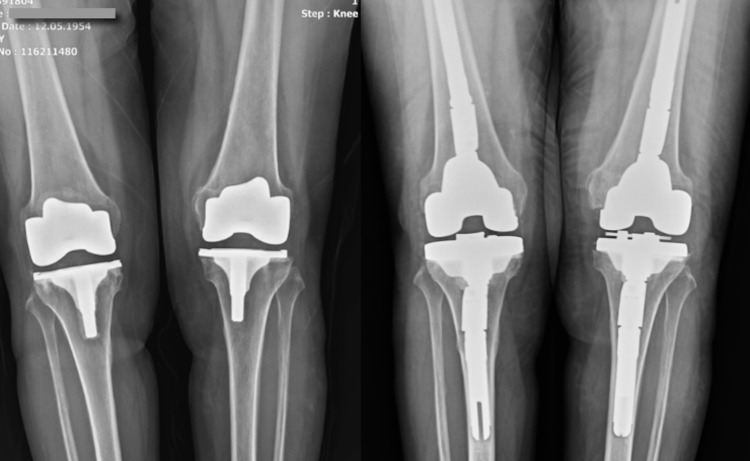
Case 2 Preoperative and Postoperative Knee X-Ray

In our second case, a 65-year-old female patient was admitted to our service due to bilateral aseptic loosening. The patient was operated by simultaneous bilateral total knee arthroplasty six years ago. Regional anesthesia could not be performed due to implants placed in the spondylolisthesis operation 10 years ago. Surgical procedures, which lasted 150 minutes, were repeated as in the first case for the patient undergoing general anesthesia. The patient needed two units of ES replacement. Due to her comorbid diseases and general anesthesia, the patient was taken into the intensive care unit postoperatively and was returned to the ward and mobilized on postoperative Day 2 (Figure 3).

Case 3

**Figure 4:**
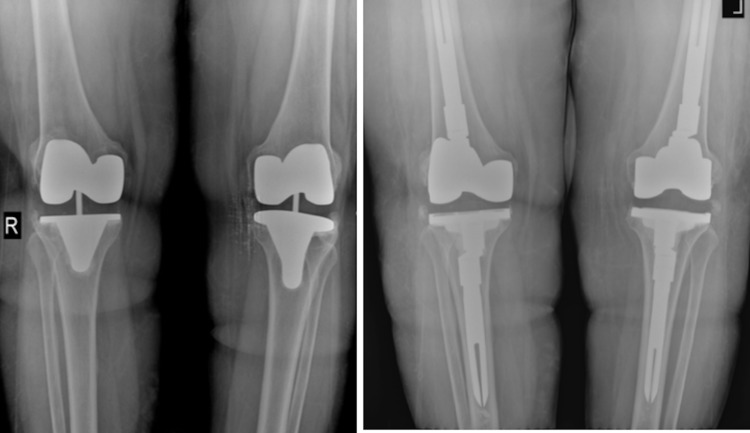
Case 3 Preoperative and Postoperative Knee X-Ray

Our third case was a 59-year-old female patient who was operated on the right knee six and on the left knee four years ago. The patient had a history of needing intensive care unit support due to pneumonia after primary knee arthroplasty surgeries. It was taken into consideration that each operation poses a separate risk for the patient and bilateral implementation was decided. One unit of ES replacement was given intraoperatively. The operation lasted for 200 minutes, without deterioration in the general condition of the patient who did not need postoperative intensive care (Figure 4).

Case 4

**Figure 5:**
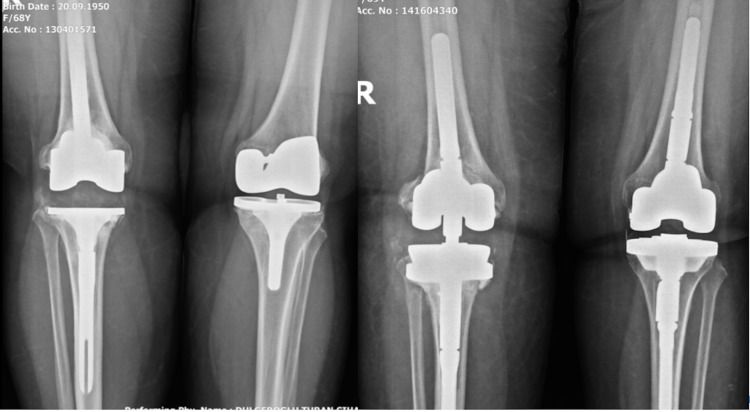
Case 4 Preoperative and Postoperative Knee X-Ray

Our fourth case, a 68-year-old female patient was operated by simultaneous bilateral primer total knee arthroplasty 12 years ago. The patient had a two-stage revision knee arthroplasty due to septic relaxation in her right knee six years ago. The patient was admitted to our service due to bilateral aseptic loosening. The operation lasted about 130 minutes. One unit of erythrocyte suspension (ES) replacement was given in response to bleeding in the patient, who did not develop any complications during the operation. PCA application was performed via an epidural catheter for postoperative pain. An intra-articular transamine application was performed. In the first 24 hours, the patient needed two units of ES replacement (Figure 5).

## Discussion

In our case series of four patients, the single-staged procedure resulted in short hospital stay, low hospital bills, and uncomplicated operation.

Studies comparing unilateral and bilateral primary TKA report different results. Although there have been several publications in the literature regarding unilateral or bilateral primary TKA; only a few studies have assessed bilateral revision TKAs. In a single session in selected patient groups, consecutive bilateral TKA applications were found not to increase complication rates. Likewise, two-sided revision TKA can be performed in two phases or at the same time as a one-step procedure. The decision to perform a simultaneous bilateral revision TKA is controversial because of the complication rates and because of the few case series reported. Very few cases have been reported in the literature on the subject. In the literature, Lonner et al., in a retrospective study of patients with 49 bilateral simultaneous revision TKA operations, showed that there were no postoperative cardiovascular complications, stroke, or death detected [7]. They had predicted minor complications, however, these were deemed to be temporary.

In the case reported by Vaishya et al., a 67-year-old patient with TKA applied 14 years ago had a bilateral revision TKA in the same session [8]. The postoperative periods of the patient were without adverse effects, with full functioning and without the symptoms of loosening being reported on the stable knee.

In our study, we also applied bilateral revision TKA in the same session in all four of our cases and reported short-term knee replacement results, Lysholm knee scores, complications, blood transfusion needs, and cost statistics. We evaluated postoperative pain using the Visual Analog Scale (VAS) at 24 and 48 hours.

The potential advantages of performing simultaneous bilateral revision TKA may be considered as reduced hospital stay and costs; single application of anesthetic drugs; and a single rehabilitation process. Bilateral rehabilitation of both knees at the same time may also prevent further deterioration of the contralateral knee prosthesis due to excessive weight bearing after unilateral revisions.

## Conclusions

Simultaneous bilateral revision TKA procedures for selected patients may provide a reduced risk of anesthesia, shorter stay in hospital, and lower surgical costs as compared to two separate revision surgeries for each knee. Bilateral revision TKA surgeries on correct patient selection is a surgical procedure that can be performed safely by an experienced team.
